# Temporally auto-correlated predator attacks structure ecological communities

**DOI:** 10.1098/rsbl.2022.0150

**Published:** 2022-07-06

**Authors:** Sebastian J. Schreiber

**Affiliations:** Department of Evolution and Ecology, and Center for Population Biology, University of California, Davis, CA 95616, USA

**Keywords:** apparent competition, predation, storage effect, coexistence, priority effects, environmental stochasticity

## Abstract

For species primarily regulated by a common predator, the P* rule of Holt & Lawton (Holt & Lawton, 1993. *Am. Nat.* **142**, 623–645. (doi:10.1086/285561)) predicts that the prey species that supports the highest mean predator density (P*) excludes the other prey species. This prediction is re-examined in the presence of temporal fluctuations in the vital rates of the interacting species including predator attack rates. When the fluctuations in predator attack rates are temporally uncorrelated, the P* rule still holds even when the other vital rates are temporally auto-correlated. However, when temporal auto-correlations in attack rates are positive but not too strong, the prey species can coexist due to the emergence of a positive covariance between predator density and prey vulnerability. This coexistence mechanism is similar to the storage effect for species regulated by a common resource. Negative or strongly positive auto-correlations in attack rates generate a negative covariance between predator density and prey vulnerability and a stochastic priority effect can emerge: with non-zero probability either prey species is excluded. These results highlight how temporally auto-correlated species’ interaction rates impact the structure and dynamics of ecological communities.

## 1. Introduction

Predation or resource limitation can regulate populations. When multiple species are regulated by the same limiting factor, long-term coexistence is not expected under equilibrium conditions. Regulation due to a common, limiting resource can result in the R* rule: the species suppressing the resource to the lower equilibrium level excludes other competitors [1–3]. Regulation due to a common predator can result in the P* rule: the prey species supporting the higher equilibrium predator density excludes the other prey species [4–6]. Yet, many coexisting species share a common resource or a common predator. Understanding mechanisms permitting this coexistence is central to community ecology. One of these coexistence mechanisms, the storage effect for competing species, relies on temporal fluctuations in environmental conditions [7–9]. Whether an analogous, fluctuation-dependent mechanism exists for species sharing a common predator is studied here.

Similar to species competing for a common resource, species sharing a common predator can exhibit mutually antagonistic interactions [10]: increasing the density of one prey species leads to an increase in predator density and a resulting increase in predation pressure on the other prey species. Thus, to the uninformed observer, the prey appear to be competing. Empirical support for apparent competition is extensive [4,11,12] and has significant implications for conservation biology [13]. When the shared predator is the primary regulating factor, Holt & Lawton [5] demonstrated that one prey excludes the other via the P* rule. Yet in nature, coexisting species often share common predators. Holt & Lawton [5] found that spatial refuges, resource limitation and donor-controlled predation could help mediate coexistence. However, environmentally driven fluctuations in demographic rates did not promote coexistence [5,14]. These studies, however, assumed environmental fluctuations are temporally uncorrelated.

By contrast, environmental fluctuations are known, both theoretically and empirically, to mediate coexistence for species competing for a common resource. In a series of influential papers [7,8,15,16], Chesson identified two fluctuation-dependent coexistence mechanisms: nonlinear averaging and the storage effect. Empirical support for these mechanisms exist in a diversity of systems [9,17–22]. A key ingredient for the storage effect is a positive covariance between favourable environmental conditions and species’ densities. Temporal auto-correlations, which are commonly observed in environmental factors [23,24], can generate this positive covariance [25].

Here, temporally auto-correlated fluctuations in demographic rates are shown to mediate coexistence of prey species primarily regulated by a predator and to generate stochastic priority effects. To derive these conclusions, stochastic models of predator–prey interactions are studied using a mixture of analytic and numerical methods.

## 2. Model and methods

Following Nicholson & Bailey [26] and Holt & Lawton [5], the model considers two prey species with densities *N*_1_, *N*_2_ that are regulated by a common predator with density *P*. In the absence of predation, the density of prey *i* increases by a factor, its finite rate of increase *R*_*i*_(*t*), in generation *t*. Individuals of prey *i* escape predation with probability exp (−*a*_*i*_*P*) where *a*_*i*_ is the attack rate on prey *i*. Captured individuals of prey *i* are converted to *c*_*i*_ predators. To ensure population regulation, predators immigrate at rate *I* > 0 [5,14]. Allowing for fluctuations in the demographic rates, the model becomes

2.1 $$\begin{array}{rl} N_{i}(t+1) & =N_{i}(t)R_{i}(t)\exp(-a_{i}(t)P(t))\;\text{ with }\;i=1,2\\ \mathrm{and}\;P(t+1) & =\sum_{i=1}^{2}c_{i}(t)N_{i}(t)(1-\exp(-a_{i}(t)P(t)))+I(t). \end{array}\}$$

Consistent with meteorological models [27,28], fluctuations in logarithmic demographic rates are modelled as a first-order auto-regressive processes (see electronic supplementary material, (A1) in appendix). For example, the log attack rates ln *a*_*i*_(*t*) are characterized by their means $\bar{\ln\;a_{i}}$, their variances $\sigma_{i}^{2}=\mathrm{Var}[\ln a_{i}(t)]$, their temporal auto-correlation *ρ* = Cor[ln *a*_*i*_(*t*), ln *a*_*i*_(*t* + 1)] and their cross-correlation *τ* = Cor[ln *a*_1_(*t*), ln *a*_2_(*t*)]. For the numerical simulations, the auto-regressive processes are Gaussian, i.e. the attack rates are lognormally distributed.

The dynamics of (2.1) are explored using analytical and numerical methods. The analytical methods rely on the invasion growth rates (IGRs) of the prey that correspond to the average growth rates when the prey species becomes rare [7,29–32]. When both IGRs for the prey are positive, both species increase when rare and coexist. When both IGRs are negative, there is a stochastic priority effect, i.e. with non-zero probability either prey species is excluded. When the IGRs have opposite signs, the species with the positive IGR may exclude the other species. Analytical approximations for these IGRs are derived for small environmental fluctuations and computed numerically using R (details in electronic supplementary material, appendix).

## 3. Results

The invasion growth rates (IGRs) of the prey species are defined by assuming one species, say species *j*, is common (the resident) and the other is infinitesimally rare (the invader), say species *i* ≠ *j*. The resident prey and predator species are assumed to coexist (see electronic supplementary material, condition (A2) in appendix) and have reached a stationary distribution. Let *P*_*j*_(*t*) be the predator densities at this stationary state. At stationarity, the average intrinsic growth rate of prey *j* equals the average predation rate:

3.1 $$\bar{\ln R_{\;j}}=\bar{a_{\;j}P_{\;j}}.$$

When introduced at (infinitesimally) low densities, the IGR of prey *i* equals the difference between its average intrinsic growth rate and its average predation rate (see electronic supplementary material, (A5) in appendix)

3.2 $$r_{i}=\bar{\ln R_{i}}-\bar{a_{i}P_{\;j}}.$$

We use equations (3.1) and (3.2) to show the P* rule holds when *a*_*i*_(*t*) are temporally uncorrelated even if the other demographic rates are auto-correlated. Then we show how auto-correlations in *a*_*i*_(*t*) generate alternative ecological outcomes.

### (a) Temporally uncorrelated attacks and the P*-rule

If the attack rates are temporally uncorrelated (*ρ* = 0), then the average attack rate of the resident prey equals the product of the average attack rate and the average predator density: $\bar{a_{\;j}P_{\;j}}=\bar{a_{\;j}}\bar{P_{\;j}}$. Consequently, equations (3.1) and (3.2) imply prey *i*’s IGR is proportional to the difference in the average predator densities supported by prey *j* and prey *i*, respectively:

3.3 $$r_{i}=\bar{a_{i}}\times (\bar{P_{i}}-\bar{P_{\;j}}).$$

Hence, if prey 1 supports the higher predator density ($\bar{P_{1}}>\bar{P_{2}}$), then *r*_1_ > 0 and *r*_2_ < 0 and prey 1 excludes prey 2. The opposite conclusion holds if prey 2 supports the higher predator density ($\bar{P_{2}}>\bar{P_{1}}$).

### (b) The resident and invader attack covariances

**Figure 1.**
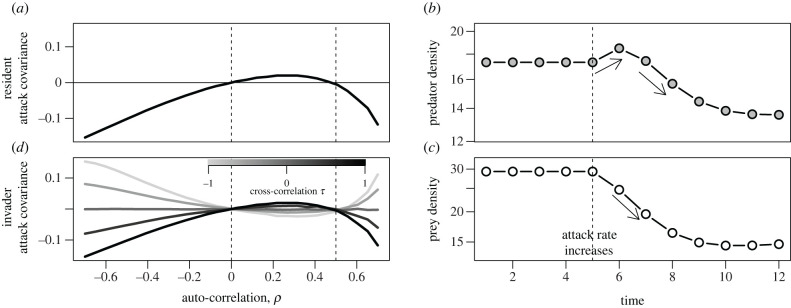
Resident and invader attack covariances depend on auto-correlations in a nonlinear fashion. In (*a*), the resident attack covariance Cov[*a*_*j*_, *P*_*j*_] is a function of *ρ* = Cor[ln *a*_*j*_(*t*), ln *a*_*j*_(*t* + 1)]. Dashed lines correspond to analytical predictions of where this covariance vanishes. This nonlinearity stems from the short-term versus long-term effects of an increase in the predator attack rate on the predator (*b*) and prey (*c*) densities. In (*d*), the invader attack covariance Cov[*a*_*i*_, *P*_*j*_] is plotted for different cross-correlations *τ*. Parameters: ${\bar{R}}_{1}={\bar{R}}_{2}=2$, $\bar{\;\;I\;}=10$, $\bar{\ln a_{1}}=\bar{\ln a_{2}}=\ln0.025$, $\sigma_{i}^{2}=1$ and ${\bar{c}}_{1}={\bar{c}}_{2}=1$.

Unlike the temporally uncorrelated environments, auto-correlated predator attack rates generate a covariance Cov[*a*_*j*_, *P*_*j*_] between the predator attack rates *a*_*j*_(*t*) and the predator densities *P*_*j*_(*t*) when prey *j* is the resident. This resident attack covariance depends on the auto-correlation *ρ* of *a*_*j*_(*t*) in a nonlinear fashion (figure 1*a*). An analytical approximation (see electronic supplementary material, equation (A8) in appendix) shows the resident attack covariance is negative when either the auto-correlation *ρ* is negative (*ρ* < 0) or *ρ* is greater than the reciprocal of the prey’s mean finite rate of increase ($1/{\bar{R}}_{\;j}<\rho <1$). Only for positive, but not too positive auto-correlations ($0<\rho <1/\bar{R_{\;j}}$) is the resident attack covariance positive.

Figure 1*b*,*c* provides a graphical representation of this non-linear relationship. When the predator’s attack rate increases, there is a short-term increase in the predator’s density. However, the continual decrease in the resident prey’s density ultimately results in a reduction in the predator density. When the auto-correlation is sufficiently positive, increases or decreases in attack rates persist for a long time and, thereby, generate a negative resident attack covariance. Less positive auto-correlations or negative auto-correlations play out on shorter timescales and generate positive or negative covariances, respectively.

Temporally auto-correlated attack rates on prey *i* (the invader) also generate a covariance between their attack rates *a*_*i*_(*t*) and the predator densities *P*_*j*_(*t*) (figure 1*d*). The sign of this invader attack covariance Cov[*a*_*i*_, *P*_*j*_] is determined by the cross-correlation *τ* = Corr[ln *a*_*i*_, ln *a*_*j*_] between the attack rates. When this cross-correlation is positive, the invader and resident attack covariances have the same sign (two darkest lines in figure 1*d*). When this cross-correlation is negative, these two covariances have opposite signs (two lightest lines in figure 1*d*).

### (c) Auto-correlated attack rates alter ecological outcomes

**Figure 2.**
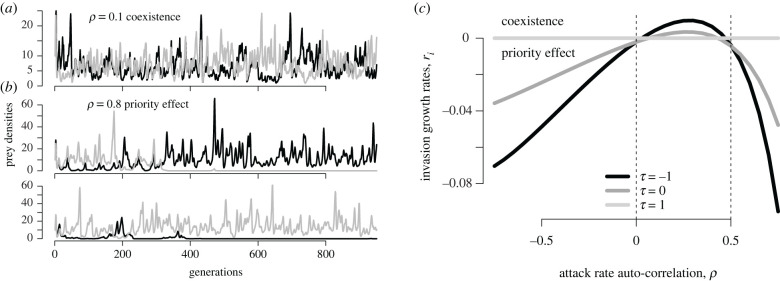
Auto-correlated attack rates alter ecological outcomes. In (*a*), the dynamics of coexisting prey species and (*b*) two realizations of the dynamics of prey species exhibiting a stochastic priority effect. In (*c*), the invasion growth rates *r*_*i*_ for both prey species when rare as a function of the temporal auto-correlation *ρ* in attack rates. Different lines correspond to different cross-correlations *τ* = −1, 0, 1 in the attack rates. Dashed lines correspond to where the analytic approximation of IGRs *r*_*i*_ are zero. Parameters: ${\bar{R}}_{1}={\bar{R}}_{2}=2$, $\bar{I}=10$, $\bar{\ln a_{1}}=\bar{\ln a_{2}}=\ln0.04$, $\sigma_{i}^{2}=0.25$, ${\bar{c}}_{1}={\bar{c}}_{2}=1$ and *τ* = 0.5 for panels (*a*,*b*).

Owing to their effect on the attack covariances, auto-correlated attack rates can alter ecological outcomes (figure 2*a*,*b*). This impact is best understood when the prey only differ in the timing of predator attacks (i.e. *R*_1_ = *R*_2_, $\bar{\ln a_{1}}=\bar{\ln a_{2}}$, $\sigma_{1}^{2}=\sigma_{2}^{2}$ but *τ* = Corr[ln *a*_*i*_, ln *a*_*j*_] < 1). Then the IGR of prey *i* equals the difference between the resident and invader attack covariances:

3.4 $$r_{i}=\mathrm{Cov}[a_{\;j},P_{\;j}]-\mathrm{Cov}[a_{i},P_{\;j}].$$

Whenever the prey species experience differential predation in time (*τ* < 1), the sign of the IGR *r*_*i*_ is determined by the resident attack covariance (figures 1*a*,*d* and 2*b*). Hence, if the auto-correlation *ρ* in the attack rates is positive but not too positive ($0<\rho <1/\bar{R}$, dashed lines in figure 2*c*), the IGRs are positive for both species and the prey coexist (figure 2*a*). Alternatively, if the auto-correlation *ρ* is negative or too positive (*ρ* < 0 or $\rho >1/\bar{R}$), then IGRs are negative for both species and the prey exhibit a stochastic priority effect (figure 2*b*).

More generally, when the prey species differ in their intrinsic fitness *R*_*i*_ and differ in their mean attack rates, the invasion growth rate of the prey *i* depends on these differences:

3.5 $$\begin{array}{r} r_{i}=\bar{\log R_{i}}-\bar{\log R_{\;j}}+(\bar{a_{\;j}}-\bar{a_{i}})\bar{P_{\;j}}+\mathrm{Cov}[a_{\;j},P_{\;j}]-\mathrm{Cov}[a_{i},P_{\;j}]. \end{array}$$

The first term $\bar{\log R_{i}}-\bar{\log R_{\;j}}$ corresponds to the difference in the mean intrinsic rates of growth of the two prey species. When prey species *i* has a larger mean intrinsic rate of growth, this term is positive, otherwise it is negative. The second term, $(\bar{a_{\;j}}-\bar{a_{i}})\;\bar{P_{\;j}}$, is proportional to the difference in the mean attack rates. When the common prey species is less vulnerable on average (i.e. $\bar{a_{i}}<\bar{a_{\;j}}$), this term is positive, otherwise it is negative. The final pair of terms, the difference in the resident and invader attack covariances, is equivalent to (3.4). Hence, differences in the prey’s mean intrinsic rates of growth or mean vulnerability to predation can alter the effects of temporally auto-correlated attack rates. For example, large differences in mean attack rates can lead to exclusion despite the attack covariances helping increase IGRs.

## 4. Discussion

For species primarily regulated by a common predator, coexistence is not expected under equilibrium conditions: the prey supporting the higher predator density can exclude the other via apparent competition [5,14]. Similar to the storage effect for competing species [7,33], we found that environmental fluctuations impacting prey specific attack rates can modify this ecological outcome. Two conditions are necessary for these alternative outcomes. First, fluctuating environmental conditions must differentially impact the predator attack rates on the different prey species. This condition is equivalent to ‘species-specific responses to environmental conditions’ required for the storage effect [7]. The second condition requires a non-zero, within-generation covariance between predator attack rates and predator density. This attack covariance is analogous to the ‘environment–competition covariance’ of the storage effect for competing species [7]. When positive, the attack covariance results in relatively lower predation rates on prey that become rare and, thereby, facilitates their recovery from low densities. Hence, coexistence is more likely. When the attack covariance is negative, predation rates are relatively higher on prey that become rare resulting in a stochastic priority effect.

There is empirical evidence that suggests both conditions are likely to occur in nature. For the first condition, differences in prey vulnerability to predation provide multiple pathways for generating asynchronous attack rates among multiple prey [34]. These pathways include differences in micro-habitat and refuge availability [35,36], environmental stressors [37], phenology [38] and morphology and behaviour [39–42]. For the second condition, temporal auto-correlations are ubiquitous in environmental factors that drive these pathways [23,24,43] and, as shown here, can generate covariances between attack rates and predator densities.

We demonstrate that both the sign and magnitude of the temporal auto-correlations in attack rates determine the sign of the attack covariance. For positive auto-correlations, the sign of this covariance depends on the timescale at which the fluctuations occur. When temporal auto-correlations are weak, fluctuations occur on shorter timescales, generate a positive attack covariance, and promote coexistence. By contrast, when temporal auto-correlations are strong, fluctuations occur over longer timescales, generate a negative attack covariance, and promote stochastic priority effects. Similarly, for continuous-time models of species competing for a common resource, Li & Chesson [44] found that fast resource depletion generates a positive environment–competition covariance and, thereby, can promote coexistence. This positive environment–competition covariance arises from consumer attack rates being positively auto-correlated at the timescale of the resource depletion.

The work presented here and earlier work [25,44] highlight that covariances between species densities and *per capita* species interaction rates can fundamentally alter the composition and dynamics of ecological communities. These covariances can be driven by the sign and magnitude of auto-correlated fluctuations in environmental conditions. Importantly, the magnitude of these auto-correlations can lead to different ecological outcomes due to differences in transient versus long-term responses of species to changing interaction rates [45,46]. Understanding how these effects combine across multiple species, how they interact with other coexistence mechanisms [33], and how they are impacted by demographic stochasticity [47–49] provide significant challenges for future work.

## Data Availability

The data are provided in the electronic supplementary material [50].
